# Hypodermic Medication

**Published:** 1883-05

**Authors:** Wm. H. Coggeshall

**Affiliations:** Richmond, Va.


ATLANTA

### MEDICAL REGISTER.

VOL. II.] MAY, 1883. [No. 8

### Original.

### HYPODERMIC MEDICATION.

BY WM. H. COGGESHALL, M.D., RICHMOND, VA.

For convenience of use, smallness of dose, utility in emergency, affording rest and respite to the digestive organs, the invention and employment of hypodermic means of medication have created a revolution in therapeusis. Before such discovery the possibilities of giving medicine in any manner save *per orem*, were either by absorption through the rectum; by inunction; or by displacing the cutis by blisters and sprinkling the medicine in powder upon the denuded surface of *dema vera*, known as the endermic method. Each of these had, and even now has its particular necessity of use, although with the exception perhaps of the last mode, the slowness of action of the medicine used prevents its acceptance in cases where a rapid effect is required. In the use of the endermic method, besides the pain and discomfort produced, which are so unpleasant to the patient, the destruction of the outer layer of the skin leaves a raw surface, whose action in absorption is always attended with a certain degree of uncertainty, and the results following the use of a medicated powder thereon, are often disproportioned to the efect desired. The most successful use of that method that has occurred in my experience, has been in two or three cases of functional or reflex vomiting, where the blister alone has failed to produce the desired effect, and quietude of the stomach has only been restored by dusting a powder of morphia thinly over the denuded surface. In the older textbooks this endermic method of therapeusis was recommended in various diseases, but I am inclined to doubt, if, before the active principles or alkaloids of the different drugs were isolated, there was much benefit to be derived from the use of the powdered roots, etc., then employed, when applied in this manner. The discovery of the means of introducing soluble medicines into the circulation soon deprived this endermic mode of all favor and with the exception of an occasional use of the sulphates of quinia and morphia in that way the busy practitioner has almost entirely ceased to employ such method of medication.

During my student life I required of more than one of my perceptors and since at different times have broached the subject in conversation with practicing physicians as to the name of the inventor, and have often been surprised to find so little known of the early history of the hypodermic syringe. In consequence, it seems to me that a few words on the subject may not be found unwelcomed.

First of all a thought upon the stymology of the word "hypodermic." It is manifestly incorrect, as it is derived from *hypo* (under) and *derma* (skin), and reasoning by analogy, at least, we should form the word "hypodermatic," as the same rule has held good throughout the formation of our language, as instanced by the words spermatic, traumatic, rheumatic, dagmatic, etc. But as "hypodermic" has held its present form so long, and entered into our vocabulary so generally, I presume it will be likely to hold its own; and so shall therefore in the following pages write the word according to ordinary usage.

The first use of the subcutaneous method of administering medicine that I have been able to find account of was in 1843, by Dr. Rynd, of Dublin. He, however, did not make public mention of it for a space of ten years, although he claims to have practiced it more of less during that period. The immediate cause of his giving the matter publicity was the publication in the *Edinburg Medical and Surgical Journal* of an article by Dr. Alex. Wood, of that city, describing his method of incising the skin and subcutaneous cellular tissue, and injecting soluble medicines into it by means of a finely pointed syringe. Upon investigation of the claims of both gentlemen, it became known that the same original idea had been first acted upon by both at about the same period, namely, in 1843. There has been a claim of priority in this matter made by Kurzak and his friends of Vienna, but I have after a long search been able to find no proof of it myself. In this country two medical gentlemen of New York city, Drs. J. E. Taylor and Washington, have made claim that they used almost precisely the same method as early as 1839, and although they did not make mention of the fact until many years had passed after the honors of discovery had been granted Drs. Rynd and Wood, yet the testimony of disinterested practitioners of their city, who testify that they saw the gentlemen named make use of the practice often between the years 1840-50, is enough to satisfy us that the use of the method was original with them, but that they failed to realize the importance of the matter in not giving publicity to what they were doing. We may fairly reckon Drs. Wood and Rynd the inventors of the method, as they were first to call attention to a syringe made specially for the purpose of subcutaneous injection. The needle on their syringes, however, was large, and the whole instrument was awkward and clumsy, but the germ of the hypodermic syringe was there, and it sufficed for the little use it had until Chas. Hunter, surgeon, of London, turned his attention to it. To him belongs the great credit of not only improving it, and making it the delicate means of medication it now is, but of taking deep interest in the subject of hypodermic medication generally; and by careful and diligent work in speaking and writing, he aroused the profession to the great value of the syringe, and not content with the energy and zeal exhibited in his own country he forced upon the recognition of the medical men of France and Germany the utility of the method; making special visits to those countries for that purpose. To Charles Hunter more than to any other one man are we indebted for the advantages we derive from the proper use of the hypodermic syringe, and if we accept the dictum of Sidney Smith, that "He is not the inventor who first says the thing, but he who says it so long, so loudly, and so clearly that he compels mankind to hear him," then to Mr. Hunter the greatest honor in this matter should be accredited.

The first to use the real instrument in this country was that eminent teacher to whom the profession owes so much in other ways, Dr. Fordyce Barker, of New York, who brought one with him on his return from England in 1856. It was received with cordiality by the practitioners of his city, and it is hardly necessary to state to what an extent its use has grown. A physician without his hypodermic syringe to-day is an anomaly, only to be compared to a soldier without his bayonet; he is still serviceable at long range, but in a hand to hand conflict he is quite likely to be worsted. Instrument makers have changed, improved, and remodeled the syringe, but of all the thousand styles and modifications, the plain fenestrated model of Hunter's invention is still the best for general use. The delicate hollow needle, the sharp point, and the open eye on the side just below the point will probably never be improved upon for that purpose.

The purpose of this paper being to briefly discuss the different medications by hypodermic means, it has seemed to me hardly worth while to say much about such use of morphia. The ordinary use of the drug is well understood, and commonly practiced, and to do justice to all branches of the subject of its use and abuse would occupy more space than the limit of this paper allows, so that throughout the remainder of the article I shall only mention it incidentally.

The peculiarities of different patients in reference to hypodermic injections are well known; one person being affected by a dose that would be considered a placebo by another, and the necessity of remaining firm while a needle is pushed beneath the cutis requires almost as great a courage on the part of one patient, as would be required for the leading of a storming party by another. There seems, also, to be a dissimilarity in the degree of inflammation produced in different individuals by the mere subcutaneous introduction of a liquid. Occasionally we meet with a patient in whose tissues even the injection of pure water will light up a plelegmon which may not be easily controlled, while generally a careful introduction of any non-irritant solution is quietly borne. There is no doubt, however, but that in certain cases, a peculiar condition of the system will at times cause an inflammation of the parts resulting perhaps in abcess, from an injection, which given at a period of general health, is followed only by the usual slight soreness we are accustomed to notice. It is a common belief that a bypodermic solution of any kind, will, after a certain degree of age is attained, produce serious inflammatory results upon use, but the experience of Dr. Wm. H Thompson in his service at Roosevelt Hospital, New York, city, would seem to entirely negative the truth of that impression. Dr. Taylor, his house physician in 1882, states that during the fourteen months he was interne there, there were given to patients over three thousand hypodermic injections of different substances, such as magendie's solution, morphia and atropia, atropia, Leute's solution of quinine, whisky, digitaline and whisky, ether, carbolic acid, etc., and that in no case was the injection of any solution followed by abcess. He further states that there was only one case followed even by inflammatory induration, and that one seemed to depend upon individual idiosyncracy, as succeeding trials upon the patient with different needles, and solutions, produced the same result. The notable portion of his report I give in his own words: "Our solutions are not always fresh, and often contain confervæ, and there is no special pains taken to clean the needles. Solutions over a month old and full of confervære have frequently been used."*

I know that the above experience is opposed to the general belief on the subject, and I must confess that I am

*New York Medical Record*, August 26, 1882.

myself afraid to follow even in the footsteps of so illustrious a teacher as Prof. Thompson in this regard, but on the contrary endeavor to take the precautions necessary to avoid any formation of ferment. The first and most reliable, is of course, to make a fresh solution when an injection is required, but there occasionally comes a time when we desire to make up more than we can use at once, and in such cases the addition of gr.ss of salicylic acid, or mins.ij of carbolic acid, to the ounce, will serve to keep the solution clear for several weeks. The tablets for hypodermic use, put up by several of the drug manufacturers, have seemed to answer well in some hands, and I think if the simple precaution is taken of warming the solution after the tablet is dissolved, say in a teaspoon held over a lamp flame or gas jet, much of the complaint that a perfect solution can not be obtained will be avoided.

It is sometimes surprising to find how small a dose of medicine administered hypodermically is required to produce given results in certain individuals. I have seen $\frac{1}{12}$ gr. of morph. sulph. cause alarming narcosis, given in a case of abnormal colic, although the general rule is that a patient in extreme pain will tolerate more than the ordinary dose.

One of the disputed points in hypodermic morphia medication, is, whether the insertion of the drug directly over or near the seat of pain is productive of better or quicker results, than such introduction at a distant part, leaving the effect to be produced through the general circulation. One would suppose such a question easily decided by careful observation, but the matter has never yet been definitely laid at rest. Such leaders in our profession as Alex. Wood, Eulenberg, Bihier, Morehouse, Chouppe, and others, believe in the doctrine of localization, while Anstie, DeRenzi, Chas. Hunter, Luton, Ruppaner, Bartholow, and I believe the great majority of the profession are generally opposed to this opinion. There are not a few however even of those who assume this negative side of the question, who believe that there are certain cases of severe neuralgia, notably those in which the nerve disturbance is entirely local, as in sciatica, where local injections are measurably to be preferred; and it is often found in practice that the patient whose necessities require a series of subdermal medications, will insist upon the injection being made as near as possible to the diseased part, especially if given for the relief of pain.

I have noticed that different authorities disagree somewhat as to the degree of sensibility of certain portions of the body to the action of medicine administered hypodermically, some like Bartholow, being "unable to observe any difference in the rapidity of the effect as influenced by the site of puncture;"* but in the main the conclusions arrived at from the experiments of Dr. H. H. Kane in reference to the rapidity of absorption in different parts are generally accepted as correct. He says, "I have found absorption from the groin and inner side of arm to rank first in point of rapidity; forearm next, leg next, abdomen next, and the thick tissues of the back last."†

The place usually reccommended for general hypodermic injection is on the arm just below the shoulder, although for convenience a point a little lower down, over the biceps muscle, is often taken for the puncture, and every practitioner has his favorite place for the initial injection.

It is interesing to notice the various opinions of successful practitioners on the subject of deep or shallow hypodermic medication. One believes that his only cases of induration or abscess, arises from injections that have been confined to the tissues directly beneath the skin, and that, given care in choosing instrument and solution, there is a total avoidance of such result if his plan is followed of thrusting the needle point into the muscular tissue to the depth of, from one quarter, to one inch;‡ while on the other hand it is not difficult to find the physician who deems deep injections surely provocative of serious inflammation, and who claims that if his method of inserting the needle just beneath the *cutis vera* is followed, the

---

*The Hypodermic Method. Phila. 1870.

†Morphia Hypodermically. New York 1880.

‡*Vide* letters from Drs. Semple, Aylward, Logan, Dunlap, Atkinson, Walker, and others in Kane's—"Morphia Hypodermically." New York. 1880.

chance of producing abscess is extremely slight.* The truth of the matter probably is, that those physicians who are careful to keep their needles and syringes clean and free from rust, are in the habit of using none but fresh solutions, and are particular to press the point of the needle entirely through the skin, are seldom if ever troubled by the annoyances above named. Once in a while of course one is liable to meet with a patient predisposed to abscess, either from idiosyncrasy, or an unhealthy condition of his system, and in such case all the precautions one can take are of little or no avail. My practice has been to keep my syringe and needles as clean as water can make them after use, and then running a little carbolized oil through them before deposition in their case. I often rub the small brass wire that is kept in the needle when not in use, with carbolized vaseline to avoid danger from rusting, and taking ordinary precaution to prevent my solutions from becoming either acid or loaded with confervæ, I find extremely little complaint from my patients as regards either induration or abscess. Naturally the solution I find most need of is one of morphia, and I have kept it made up for several months, by the means mentioned before, namely two drops of carbolic acid to the ounce, with occasional filtration, and never have been disappointed in its use.

The danger of wounding a vein accidentally is one we were all taught to avoid ten or twelve years ago, although the result can not be as serious as was formerly supposed, for we often see in medical publications great commendation of the use of a particular medicant introduced through the venous circulation.

Great stress was formerly laid on the serious results sure to follow the introduction of air into the cellular tissue by means of the hypodermic syringe, but unless the two accidents of introducing air and puncturing a vein, should occur together, I doubt there being anything serious to apprehend.

---

* *Vide* Bartholow — "The Hypodermic Method." Phila. 1879. And letters from Drs. Bergen, Thompson, Prof. Post, and others in Kane's—"Morphia Hypodermically." New York. 1880.

The injection of air into a vein which will rapidly carry it to the heart and there cause paralysis of that organ, is a danger not to be lightly regarded ; but I have seen bubbles of air driven into the arm in connection with a hypodermic injection many times, either by a careless old physician, or an over eager young æsculapian, without unpleasant results; and several years ago I had the fortune to witness a semi-intoxicated practitioner insert the point of a needle, attached to a syringe containing nothing but air, deep into the biceps muscle, and inject into that tissue half a syringe barrel full of air, under the impression that he was medicating the patient with a weak solution of morphia, as a means of relief from severe enteric colic, and although the patient was relieved of his pain, probably through the faith that moves mountains, yet there never was any symptom of local emphysema, or inflammatory action of any kind, which we expected would follow the injection.

One of the most difficult solutions for hypodermic use to prepare perfectly, has been that of quinia. I well remember the arms and legs covered with abscesses small and large, I was accustomed to see at a New York hospital a number of years ago, where the visiting physicians were endeavoring to solve the problem of making a successful quinine hypodermic solution, and since then both in the practice of others and myself, I have witnessed the same unpleasant sights. Whenever a solution containing a superabundance of mineral acid is used, induration or abscess seems inevitable. After trying and seeing tried several different preparations of quinia, I have found the following to answer the purpose better than any others in my knowledge. The first is the old formula of Dodeuil :

| | |
|---|---|
| Quin. Sulph .................... | parts ij |
| Acid, Tartaric..................... | " j |
| Aq. Distill........................ | " xx. m. |

Administering as a dose from one-third to one half the quantity of the salt usually given by the mouth. This too is an excellent preparation recommended first, I believe by A. H. Sharp, in *Proc. Am. Pharm. Ass'n*, 1874, as follows:

℞.—Quinia Sulphat.....................grs. xv.
Acid. Lact........................... " xv.
Aq................................ f℥ j.
Sodii Bi-Sulphate....................gr. ss.

When the solution of quinia is perfect, add the bi-sulphate of sodium, and keep in tightly corked vial. I have used both, and though having abscess from neither, I prefer the latter, as there is apt to be little or no induration after the injection.

One of the most important advantages of the hypodermic syringe is its convenience in cases of poisoning, where the medical attendant is desirous of the speedy effect of an antidote; and best of all, medication by this method can be produced even during the time that an attempt is being made to relieve the stomach of its contents. In no other way can we exhibit so speedily morphia in belladonna, gelsemmium, and stramonium poisoning; atropia in opium, jabonrandi, physostigma, and strychnia poisoning; strychnia in chloral poisoning; and apomorphia in carbolic acid poisoning.

In acute attacks of neuralgia, and in fact any form of what may be called spasmodic pain, morphia alone is excellent, but combined with atropia the good effect is manifestly increased; it is often the case that relief from pain is not only rapid, but that the unpleasant after effects of morphia are reduced to a minimum. I find it best to add to one ounce of Magendie's solution, one grain of atrop. sulph., and inject five minims of the mixture as an averrage dose.

Chloral hydrate has long stood the test as a valuable prophylactic and medicant in sea sickness, and in the latter case can be most generally useful hypodermically, in doses of from five to ten grains well diluted. Its employment in tetanus by means of the hypodermic syringe is well known, and M. Ore, of Paris, finding the salt so completely paralyzing reflex action of the spinal cord, removed a necrosed astragalus, during a half hour's sleep induced by injecting a little over five drams of a solution of chloral in three times its weight in water. Its tendancy to produce abcesses when a solution of any great strength is used, however, effectually precludes us from many trials of its analgesic properties.

Hypodermic injections of strychnia in neuralgia and certain forms of paralysis are coming into general use, in hospitals especially, the strength of the solution being four grains to the ounce of water, each minim equaling $\frac{1}{20}$ gr., and the primary dosage two minims. Either the sulphate or the muriate may be used. One English physician, Dr. Wm. Carter,* has reported a case which shows what large doses of the drug can some times be tolerated. He began treating the patient with five minim injections of strych. liq. and gradually increased the dose for nearly three months, until the man was receiving twenty minims ($\frac{1}{6}$ gr.) twice a day hypodermically, and at the same time taking ten minims ($\frac{1}{12}$ gr.) three times daily by the mouth, without experiencing any of the usual poisonous sypmtoms caused by the drug. The case was one of paralysis of both arms from the pressure of crutches in the axillae, and the patient was discharged cured.

In constitutional syphilis the use of hypodermic injections of corrosive sublimate, and the albuminate of mercury, are both highly extolled, especially the former. The strength commonly used of the bichoride is one grain to two drams of water; dose from five to ten minims ($\frac{1}{24}$ to $\frac{1}{12}$ gr).

Ergotin is often very valuable hypodermically, especially in the post partum and other uterine hæmorrhages, in which its best results follow an injection directly over the abdomen, of the following solution: Ergotin 30 grs.; aqua 13 drs.; glycerin 13 drs. One dram represents a grain of ergotin, and no untoward results to the cellular tissue are likely to follow a careful injection of that quantity.

In acute rheumatism and disordered action of the heart, the tinctures of veratrum viride and digitalis have been used with good effect, injections of either are liable to cause induration, even when no more serious local effects ensue.

---

*Liverpool *Med. Chir. Journal*, July, 1882.

Apomorphia is one of our strongest sedatives, in fact its full effect is that of a depressant. It dissolves readily in water, and for general use a solution of one grain in three drams of water is to be preferred, using for an injecting dose about twenty minims, or one-ninth grain. It is of great value in maniacal delirium, especially that form proceeding from the long continued extreme imbibition of diffusible stimulants. Its action as an emetic hypodermically is exceedingly rapid, and far more certain than any other therapeutic agent that can be exhibited in the same manner.

Pilocarpine is invaluable as a sudorific, there being no medicant comparable with it in this respect, in hypodermic use. It is readily soluble in water, and most profuse sweating is induced by the injection of one-sixth to one-fourth grain, either of the nitrate or muriate. Ten minims of a solution of one grain to one dram of water is usually followed upon injection by complete diaphoresis. It is indicated of course wherever it is desirable to promote extreme perspiration, and has been used successfully in cases of diptheria, in dropsies, and in breaking the chill of malaria.

Carbolic acid has been used extensively in Germany in erysipelas, and numbers of successful cases have been reported where a two per cent aqueous solution has been employed. It has also been found valuable in cases of local pruritis. Fifteen minims of a solution of one minim or grain to a dram of water is the average German dose.

In the *New York Med. Record* for February 24th, 1883, may be found an account, copied from a continental medical journal, of three cases of muscular rheumatism, treated by a Florentine physician, who used a two per cent solution, and found the results eminently satisfactory. He injected the medicine deep into the involved muscles, and reports that at one sitting he has used as many as forty injections, containing nearly twelve minims of the acid, and in no case did he observe any of the poisonus effects of the drug. In looking over different reported instances of the use of the acid hypodermically, I can not find that in any case has there been the pain, induration, or abscess after the injection that one would naturally expect to follow it.

Amyl nitrite has been lately tried subdermally by Dr. J. J. F. Barnes, who describes his success in several cases of lumbago and colic, with a ten per cent. solution in rectified spirit, in the *British Medical Journal*, June 3rd, 1882. He used for each injection ten minims of the solution (one minim of amyl), and had no difficulty in giving speedy and permanent relief, with no suppurative symptoms at the point of injection.

Sulphuric ether has been in use as a subcuticular injection for some time in Germany, being employed there in adynamic stages of typhoid fever and pneumonia, in doses of from ten to forty minims, but though apparently of considerable constitutional value, the local inflammation in nearly every case was quite severe.

The most successful use of chloroform by the hypodermic syringe that I can find, is in cases of odontalgia, where the point of the needle is pressed in a short distance between the tooth and the gum, and two or three minims slowly injected. I have been able to allay the atrocious throbbing pains attendant upon toothache several times by this method, and have found the slight pain excited by the introduction of the needle easily borne by the patient. The injection of this liquid into the subcutaneous or muscular tissues is exceedingly apt to produce serious inflammation, and even gangrene.

Ol. Eucalyptol was used in one case of pyæmia by Dr. S. Sloan, who reported it in the *Lancet* for Sept. 2nd, 1882. He believes that the disease was cured by the treatment, and expresses the hope that the remedy will be tried sufficiently to prove or disprove its value as a cure for this formidable disease. He injected eight minims in sixteen of olive oil, (beginning with a somewhat smaller dose), and although the injections gave rise to great swelling and inflammation, he found the patient derived so much benefit from them that he persevered until the cure was complete. It would be reasonable to suppose, however, that any hypodermic injection into the tissues of a pyæmic patient would be likely to be followed by the sequelæ he mentions.

Curare, or, as it is more commonly called, woorari, by its action paralyzing the motor nerves, and leaving unimpaired those of sensation, has often been employed in cases of tetanus, in doses of from one seventh of a grain to one grain dissolved in water, and frequently with good effect. Its use in hydrophobia has sometimes been attended with success, but unfortunately its employment in this malady with negative results is often reported; and the belief that it would prove a specific in this disease has been found to rest on no sound basis, although the few instances of cure from its hypodermic use must force the physician to make trial of it, when opportunity offers.

Camphor dissolved in twice its weight of oil of sweet almonds, has been tried in adynamic conditions of the system from disease, as a stimulant, with some little success, but the soreness and inflammation attendant upon its injection have prevented an extended trial of it.

Liq. ammonia Fort. has been employed diluted with one or two parts of water (and occasionally even in full strength), in cases of suspended animation and snake bite; five to twenty minims of a fifty per cent. dilution, however, being perhaps the most often employed as an injection. Of course as a *dernier resort* in cases of collapse from anæsthesia, etc., it should be at hand ready for use, but the local consequences of the injection are almost invariably severe, inflammation and speedily forming abscess being the usual sequelæ to its subdermal use.

Cold water injected over the epigastrium has not seldom been employed successfully in cases of phthisical and nervous vomiting, and more especially is the success insured, I fancy, if the patient remains unacquainted with the precise name of the therapeutic used.

Muriate of berberina has been used with moderate success in malarial fever, and cases of enlarged spleen in doses of $\frac{1}{2}$ gr. to 2 grs. for each injection, in a weak alcoholic solution, but a sufficient trial of the salt has not yet been made, to determine its full value.

Coffee, as an injection subdermally, is an agent not likely to be often used, but Dr. James B. Garrison, of Dewitt, Ark., in 1873, proved its value in emergency; as finding himself without other antidotes at hand in a case of morphia poisoning far out in the country, he put his hypodermic syringe to such good use, that he injected a pint of strong decoction of the berry into different portions of the anatomy of his patient, with most happy result, fully antidoting the poisonous alkaloid ingested, and leaving to mark the many points of injection no abscess or inflammation. Fortunately we are not usually dependant upon a home made decoction, for quick use in such exigency, as in cases where the cerebral and cardiac stimulation of coffee is desired we can employ either coffeine in injections of one grain dissolved in water, as often as required, or Wyeth's fluid ext. of Java coffee, in half dram doses in like manner.

Colchicin in acute muscular rheumatism, according to a case reported by Dr. A. B. Hirsch, in *Med. Times*, December 2nd, 1882, has been quite successfully used. The injections were of "five minims of a one-tenth per cent. solution of Merck's colchicin," (an imported German prepation of the alkaloid), only slight local inflammation resulting. The success in this case will probably lead to other trials of this remeday.

Sulphate of magnesia has been tried in Paris, by M. Luton, as a laxative hypodermically. He used an injection of ten centigrammes, (about 1½ grs.) of the salt, in one gramme (about 15 mins.) of water, and found its action to be that of a complete purgative, with little or no resuling local inflammation, and believes it to be worthy of more extended trial.

Resorcin, the new resin, in a five per cent. solution in water, was employed successfully in four cases of erysipelas by Dr. T. F. Bogusch,* who used o. 25 c. c. (about four minims) of the solution, at each injection, and surrounded the entire inflamed region in each case with a ring of injected points. The cases required from twenty-nine to seventy injections to complete the environment of each, and in all, the spreading of the inflammatory process was immediately stopped. This was the only treatment used, and no untoward symptoms were caused by it.

*London *Med. Record*, January, 15th, 1883.

Chlorhydrate of aspidospermine, (a salt of the alkaloid of quebraco), has attracted the attention of the German practitioners, and they find its use in dyspœna attended with success, each injection containing one half grain of the salt; but as the alkaloid is only soluble in an acid solution, the inevitable resulting inflammatory action and abscess, will be apt to deter the profession generally from its frequent hypodermic use.

Spirits frumenti, good rye whisky, by hypodermic use, is one of the most valuable agents we have in cases of depression of vital powers. The many instances of shock and syncope, occurring from whatever cause, where it is impossible to revive the vital energy by means of stimulants administered *per orem*, give ample opportunity for the employment of the syringe. A dose of from one to four full injections of thirty minims each, in the arm or leg of the patient, in a case of this kind, is always of great assistance in promoting the reaction so anxiously desired. It is always well to have a hypodermic syringe and a quantity of good whisky at hand, ready for use if required, after anæsthesia, and I have known some few practioners who make it an invariable rule to inject about sixty minims of the spirit hypodermically, into the subdermal tissue of a patient just before the beginning of a chloroform inhalation. It is a striking fact, in connection with the irritating and coagulating properties of alcohol, that hypodermic injections of whisky do not produce more serious local inflammation, yet it is one of the uncommon sights to see an abscess caused by such injection. The almost invariable sequela is simply soreness or lameness of slight consequence.

It may be well to mention here the practice now in vogue in England, and in some portions of this country, of giving a full hypodermic dose of morphia ($\frac{1}{8}$ gr.), a few minutes before administering an anæsthesia. The process is varied a trifle by some practitioners, who give the alkakoid just after the beginning of the administration. By both these methods, it is claimed, that anæsthesia is more rapidly induced, with more safety, and with less unpleasant after effects, than when the anæsthetic agent is given alone.

Intravenous medication by means of the hypodermic syringe, has been recommended in various diseases, but the remedy used, will, as a rule, act as well if not introduced so directly into the circulation, and the diseases where the urgency of the case requires such strong and quick medication are generally fatal in their character, though it by no means follows that a carefully made injection into the median-cephalic or any other superficial vein is in itself dangerous. It is made so only by lack of care or the use of too potent a therapeutic agent.

The surgical uses of the hypodermic syringe are almost too many to be enumerated, from the examination of the contents of an abscess of the liver, to the transmission of an astringent injection into a hydrocele sac, and from the injection of iodic acid into the tissue of a goitre, to the deposition of carbolic acid in the centre of a hæmorrhoidal tumor. Almost the entire list of alternatives and astringents have been used for injections into superficial tumors, especially those of lymphatic origin, but the success attendant upon such treatment has not been remarkable. Like all other instruments the hypodermic syringe has its limit of use, and probably the only manner by which its present value can be greatly increased, is by the addition to the pharmacopœia of new and powerful alkaloids easily soluble in water.

